# Nerve Torsion as a Pattern of Parsonage–Turner Syndrome: Literature Review and Two Representative Cases

**DOI:** 10.3390/jcm12134542

**Published:** 2023-07-07

**Authors:** Davide Glorioso, Rita Palestini, Cristina Cuccagna, Liverana Lauretti, Luca Padua

**Affiliations:** 1 Department of Geriatrics and Orthopaedics, Università Cattolica del Sacro Cuore, 00168 Rome, Italy; 2 UOC Neuroriabilitazione ad Alta Intensità, Fondazione Policlinico Universitario A. Gemelli, 00168 Rome, Italy; 3 Department of Neuroscience, Neurosurgery Section, Università Cattolica del Sacro Cuore, 00168 Rome, Italy; 4 Department of Neurosurgery, Fondazione Policlinico Universitario “A. Gemelli” IRCCS, 00168 Rome, Italy

**Keywords:** amyotrophic neuralgia, parsonage tuner, echography, ultrasonography, nerve

## Abstract

(1) Background: Parsonage–Turner Syndrome (PTS) is a rare peripheral nerve disease characterized by different degrees of nerve impairment. The recent development of nerve ultrasound has enabled the use of new data in the diagnosis of the disease. The aim of this study is to conduct a literature review about the ultrasound evaluation of PTS and present two clinical cases that are characteristic of the disease. (2) Methods: A review of the literature from the last 10 years on the topic containing data regarding nerve ultrasound was performed. In addition, two cases of patients on whom nerve ultrasound was performed at the first evaluation and at follow-up after the indicated treatment were described. (3) Results: The results of our review show that although it is defined as plexopathy, PTS is most often a form of multifocal neuropathy. We also report the most frequently used ultrasound classification and possible prognostic correlations and report our experience with the description of two paradigmatic clinical cases. (4) Conclusions: Further studies are needed to understand the true prognostic power of each degree of nerve impairment and the possible implications in clinical practice regarding treatment indications.

## 1. Introduction

Parsonage–Turner syndrome (PTS), or neuralgic amyotrophy, was first described in the first half of the twentieth century [1]. Although it was considered a rare disease, a retrospective study in 2015 found that PTS was strongly under-diagnosed with an effective incidence of 1/1000 [2]. Parsonage et al., in a cohort of 136 patients, recognized this syndrome as characterized by acute shoulder pain followed by flaccid paralysis of the shoulder and arm muscles [1]. In subsequent years, several studies were published that described similar clinical conditions involving the anterior interosseous nerve (AIN) [3], the posterior interosseous nerve (PIN) [4], and other nerves. Van Alfen et al., in 2006, in a cohort of 246 patients, accurately described the clinical features of PTS and the extent to which the upper limb was involved [5]: PTS is a patchy disorder in which paresis more frequently occurs in the muscles supplied by the superior and middle trunks. There are various hypotheses for the predisposing factors, including infections, exercise, surgery, peripartum period, vaccinations, stress, trauma, genetic predisposition, and inflammation. Most of these factors are linked to edema and inflammation of the peripheral nerves. Surgical findings showed adhesion and increased stiffness of the fascicles, followed by constriction and localized torsion [6,7,8]. The roles of electromyography (EMG) and nerve conduction studies (NCSs) were rigorously assessed, and they are the most commonly used diagnostic techniques to detect nerve injury; several neurophysiological patterns are described, but usually, motor axonal loss is the main feature [9]. The application of EMG is a useful but invasive method, and since denervation can take up to 4 weeks in order to be fully evident, the early detection of axonal involvement may be limited to neurogenic recruitment. Furthermore, NCS has been used to determine the locations of lesions in PTS. However, in the subsequent phase of the disease, during and after the occurrence of reinnervation, the NCS parameters of the affected nerves may be in the normal range, limiting the sensitivity of this approach [10]. Classically, treatment options are limited to conservative measures, such as the early administration of corticosteroids, proper pain management, and physiotherapy to treat muscle weakness and, in case of failure to recover, a surgical approach should be considered [10]. The prognosis for PTS is generally considered favorable: the neuropathic pain attenuates within a few days/weeks and the nerve paralysis heals in the following months or years [5]. However, studies conducted on a wide range of patients showed that recovery is less favorable than previously assumed [5]. The reason why nerve regeneration is less efficient in some patients with PTS is not yet known. The introduction of imaging-based diagnostic methods, such as nerve ultrasound (NUS) or magnetic resonance of the nerve (MRI), made it possible to recognize several patterns in PTS, although, in many cases, no abnormalities were observed. The use of MRI shows signs of direct and indirect nerve distress with the debasement of a high T2 signal. Gradually, especially in patients without resolution, atrophy and fat infiltration develop, with increased T1 signals and decreased muscle mass. Additionally, the “bull’s eye sign”, which indicates the presence of constriction in patients with PTS, is also reported in the literature [11]. The pattern of nerve torsion, called hourglass, was described using NUS and surgically confirmed, and it was considered pathognomonic of PTS [12,13]. In this study, we performed a review of the literature on NUS patterns in PTS focusing on the incidence of nerve torsion, and in this paper, we describe two representative cases that emphasize the power of NUS in the detection and precise localization of PTS nerve lesions, showing two different evolutions.

## 2. Literature Review

We conducted a search of the literature from the last 10 years on the use of ultrasound assessment in PTS on PubMed, Google Scholar, and EMBASE, using the following research keywords and combining all the terms: “amyotrophic neuralgia” [MeSH Terms]), “Parsonage Tuner”, “echography” [MeSH Terms], “Ultrasonography”, “ultrasound”, “nerve”. The search was limited to English-language articles published from 1 January 2012 to 26 April 2022, and the results were not filtered. From this search, 71 articles were identified, of which 19 were duplicates. Of the remaining 52 articles, 15 were not considered after we analyzed the title and abstract. Of the 37 remaining articles, 11 were excluded after reading the full text because they were irrelevant. Here, we report the contents of papers describing a wide sample of patients or novelties, and we present the overall contents of papers in Table 1. A work published in 2015 collected the features of ultrasound of the nerve in a cohort of 14 patients with clinical diagnosis of neuralgic amyotrophy [14]. The results of this study revealed 4 degrees of nerve damage recognizable by ultrasound in patients with PTS: (I) focal, multifocal, or diffuse enlargement of nerve cross-sectional area (CSA), together with structural abnormalities, such as complete loss of fascicular structure and hypoechogenicity; (II) focal incomplete constriction of the nerve or the nerve fascicle bordered by segmental nerve enlargement (III) Focal complete constriction and hourglass-like appearance; (IV) fascicular entwinement, which, on slow cross-sectional scanning over the affected nerve segment, shows gradual 360° rotation of nerve fascicles, while on the longitudinal scans, the cross of fascicles may be seen [14]. In addition, Arányi et al. point out that while segmental or diffuse nerve enlargement associated with hypoechogenicity is a nonspecific sign of nerve pathology found in other types of neuropathy, nerve constriction and fascicle rotation with an hourglass appear to be more specific for PTS [14,15,16]. The fascicular or diffuse involvement of the pathology is still not entirely clear. In fact, Lieba-Samal et al. argue that is the essential aspects are the size of the nerve and the resolution power of the ultrasound probe. They believe that in future, for small nerves, global fascicle involvement is more likely, and that improved high-resolution probes may better differentiate affected fascicles from healthy fascicles [17]. Arányi et al. suggest searching for possible nerve lesions outside the plexus and along the entire nerve, as far as can be explored [14]. The results obtained by Van Alfen et al. strengthen the hypothesis that PTS is more often a form of multifocal fasciculitis than of plexitis [18]. Van Rosmalen et al. enrolled 50 patients with PTS and 50 healthy subjects by comparing the CSA of nerves of the healthy side with the pathological side. In patients with PTS, they found increased CSA of the median and radial nerves, even on the clinically healthy side, but they could not explain this finding. However, this was similarly observed in multifocal motor neuropathy, where even nerves not clinically involved may be altered with increased CSA [19]. Regarding the brachial plexus roots, the researchers also found less pronounced CSA enlargement in roots C5–C7 (mean increase of 16–18%) than in C6 (mean increase of 28%), suggesting diagnostic significance. However, they confirmed little involvement of the ulnar nerve and lower trunk, which can be attributed to the reduced exposure of these nerves to possible mechanical stresses. Moreover, they did not detect signs of hypervascularization in pathological nerves. This finding was probably due to the fact that US was performed long after the acute event (average 16 months). This suggests that overt and continuous nerve inflammation is not a feature of classic PTS [20]. We found discordant and doubtful opinions regarding the comparison of the diagnostic power of NUS and MRI. In fact, several studies reported optimal consistency between ultrasound and MRI findings [21], while in others, it was highlighted that MRI is uninformative compared to NUS [14]. Furthermore, regarding the comparison between NUS and MRI, Lieba-Samal et al. argue that with MRI, it is possible to detect indirect signs of denervation earlier than with NUS, as it shows the edema of denervation within 24 h of onset, while ultrasound only shows muscle atrophy after a few weeks [17]. According to the findings in our review, the ultrasound-classification system developed by Arányi et al. seems to be the most widely used. However, in most cases, these uses are in case reports or work conducted on small samples, which does not allow us to fully appreciate the validity of this classification. Enlargement appears to be the most frequently recognized ultrasound sign in patients with PTS (e.g., [15] 43%; [8] 66%; [20] 100%). However, doubts about its real diagnostic power remain unresolved. One of the reasons for this is the fact that enlargement is a nonspecific sign for PTS as it is shared with other nerve pathologies, such as entrapments, metabolic stress, etc. Another aspect to take into consideration is the lack of available data; in fact, many of the studies considered do not refer to the other ultrasound changes recognized by Arányi. This makes it complicated to understand whether these other changes were not found or not searched for. The only article that excludes with certainty the presence of other alterations is that of Gruber L and colleagues [22], which confirms that, in a sample of 14 cases and 15 controls, nerve enlargement was detected in 100% and that in no case was partial/total constriction or an hourglass-like appearance recognized. Another finding from the review is that most of the patients who were candidates for surgery showed hourglass-like appearance and fascicular entwinement (e.g., [8] 100%, [11] 100%). This leads us to hypothesize that the more severe forms of pathology and those requiring surgical treatment exhibit these two patterns more frequently. Some studies showed a correlation between “fascicular hourglass constriction” and a partial or absent spontaneous recovery [6,7,8]. Arányi et al., in 2015, provided evidence for a statistically significant correlation between NUS degrees of nerve involvement and functional recovery. In fact, according to the authors, patients with a complete focal constriction (grade III) and/or cross fascicles (grade IV) found on NUS benefit from surgical treatment compared to patients presenting only nerve enlargement (grade I), which tends towards spontaneous remission. Hence, they recognized the prognostic power of the lesions shown on NUS [14]. They also proposed the hypothesis that, in addition to the type of injury, the extent of nerve constriction is also important in establishing the prognosis. Wu et al. described a cohort of 41 patients presenting spontaneous PIN palsy with nerve constriction. They concluded that, in patients in whom no spontaneous improvement within 3 months of the onset of symptoms is observed, surgical treatment might be more effective than conservative treatment [8]. Moreover, time is another topic to discuss. In fact, very often, authors report that early diagnosis is crucial for appropriate therapeutic indication. This concept is further explored by Noda et al., who emphasized the advantages of ultrasound in conducting an immediate study of the nerve and tracing any lesions that were more likely to benefit from surgical treatment [16]. They also stressed the crucial role of preoperative ultrasound in accurately recognizing and localizing constrictions that benefit from surgical treatment. A couple of years later, including a cohort of 53 patients with PTS, Arányi et al., despite the lack of unanimity in the literature, confirmed the above-mentioned data, the complicated/absent reinnervation in a nerve with complete constriction or torsion phenomena, and the subsequent indication of surgical treatment [15]. Regarding treatment, only one article deals with the topic of NUS as a possible guide to the implementation of possible therapy. Specifically, Su PH et al. described a clinical case in which they applied, with beneficial effects, ultrasound-guided electroacupuncture treatment on a PTS patient with left-shoulder pain and muscle weakness [23]. Currently, the power of ultrasound in monitoring the effect of drug therapy remains an open question [18].

## 3. Case Reports

### 3.1. Case 1

**Figure 1:**
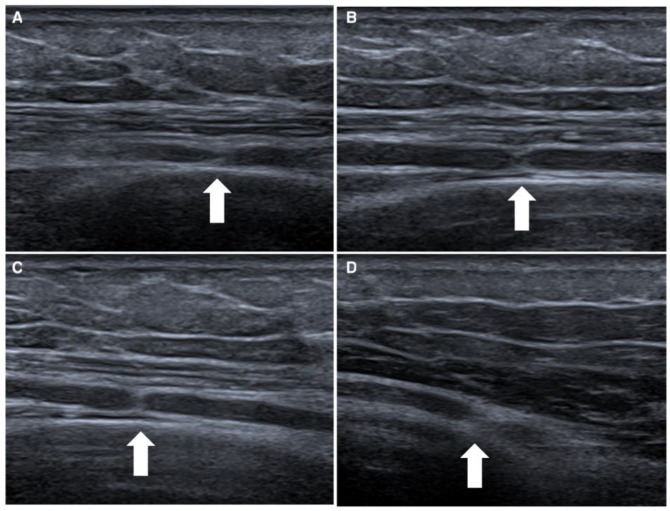
Ultrasonographic appearance of first case, a rare case of quadruple torsion of the radial nerve. In this figure is represented the radial nerve with complete constriction of four nerves, which are located 10 cm (white arrow in box (**A**)), 8 cm (white arrow in box (**B**)) and 4 cm above the elbow crease (white arrow in box (**C**)), and at the elbow crease (white arrow in box (**D**)).

**Figure 2:**
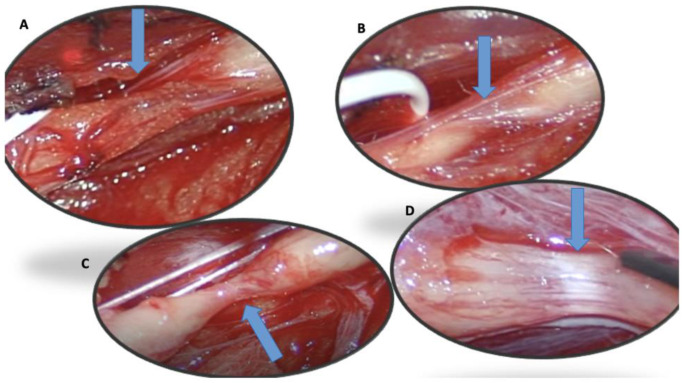
(**A**–**D**): Four torsion sites described sonographically in case 1 and confirmed surgically (blue arrow).

The first case concerns a 25-year-old male patient entering the emergency department following a fall from a kick scooter. A radiographic evaluation showed a distal-third left-clavicle fracture. It was treated conservatively with an acromion-clavear brace. In the first two weeks after the trauma, he complained of striking pain, showed decreased strength of the extensor muscles of the wrist and left-hand fingers (MRC 0), and reported paresthesia and hypoesthesia along the area of the radial nerve. Needle electromyography performed 1 month after the onset of the weakness, and denervation at rest and the absence of voluntary recruitment of the wrist and finger-extensor muscles were noted. After a month, the patient still complained of extensor-muscle plegia; therefore, a NUS was suggested. We performed NUS along the course of the radial, median, and ulnar nerves. Studying the course of the radial nerve, it was possible to visualize the focal disappearance of the physiological aspect of the nerve for about one millimeter, including torsion with complete nerve constriction at four sites: 10 cm (Figure 1A), 8 cm (Figure 1B), and 4 cm above the elbow crease (Figure 1C) and at the elbow crease (Figure 1D), immediately after the division of the deep branch from the superficial branch of the radial nerve. These impairments were clearly visible even on the long axis. Due to the presence of multiple nerve torsions with complete constriction, neurosurgical treatment was suggested. Under general anesthesia, a skin incision was performed along the course of the left radial nerve from the spiral groove to the division into the superficial branch and interosseous nerve, and the sites of torsion were pencil-marked over the skin, in accordance with the NUS findings. The radial nerve was exposed, and under magnification, the external appearance was analyzed: two of the four sites of nerve torsion were recognized on first glance (Figure 2A), while the remaining two were identified only after external neurolysis was initiated (Figure 2B): the paranevrium and the epinevrium were particularly thick and vascularized. Complete nerve straightness was obtained with the internal neurolysis (Figure 2C), but the nerve appeared to be clearly altered at the level of the previous torsions: in fact, there were two segments, corresponding to the second and to the third torsion point, in which the nerve was white and firm in consistency, signs that were suggestive of anatomical changes secondary to a neurodegenerative process (Wallerian degeneration without axonal regrowth) (Figure 2D). Net, since the nerve was unresponsive to direct electrical stimulation even distal to these clearly abnormal segments, namely at the level of posterior interosseous nerve, nerve reconstruction with an autologous graft longer than 10 cm appeared to be a hazardous procedure: we decided to assure that the nerve was in a straight route and to end the surgery. After 6 months of neurorehabilitation and neurotrophic treatment, the patient did not show any clinical or neurophysiological improvement; palliative surgery with tendon transfer will be discussed with the patient.

### 3.2. Case 2

**Figure 3:**
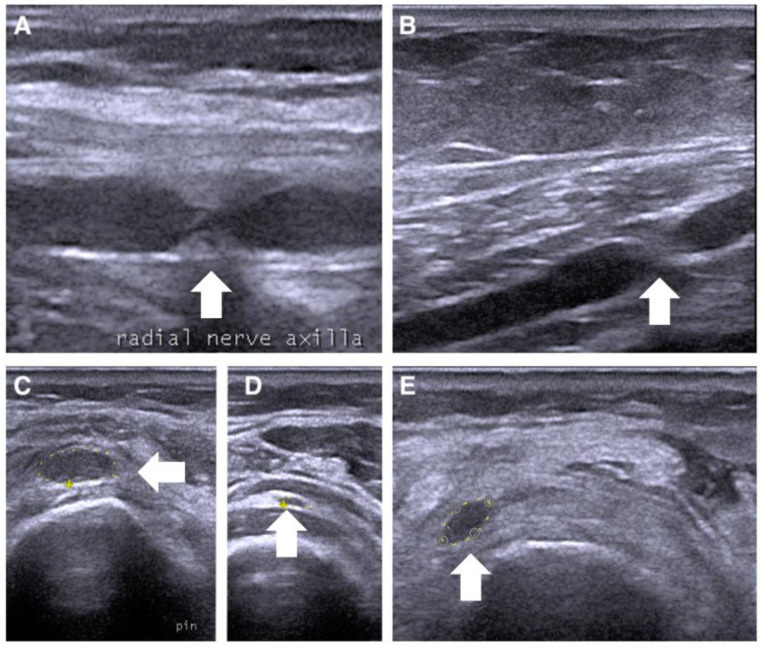
Ultrasonographic appearance of the second case of radial nerve torsion (white arrow) in at the upper arm in (**A**,**B**) on short axis, and the posterior interosseous nerve between the heads of supinator muscle (**C**–**E**) on long axis.

The second case concerns a 56-year-old female patient with left- and right-wrist fractures after a traumatic injury. During the first medical examination, a few days after the trauma, she presented striking pain and showed mild weakness of the wrist and finger flexor muscles (MRC 3) and plegia of the wrist and finger-extensor muscles (MRC 0). One month after the trauma, the patient still complained of pain and weakness. A needle electromyography showed diffuse denervation signs in the arm and forearm muscles. We also used NUS to study the course of the radial, median, and ulnar nerves. Along the course of the radial nerve, it was possible to underline the presence of a double torsion in the axilla (Figure 3A) and above the elbow (Figure 3B), with suffering of the interosseous nerve, which appeared to be increased in the cross-section area (CSA) (13 mm^2^) (Figure 3C). These nerve constrictions were visible even on the long axis. Rest and neurotropic supplements were indicated, and it was recommended that a new ultrasound evaluation be performed after 4 months. During the follow-up, a new NUS was performed along the course of the radial nerve. It was possible to visualize the complete recovery of the physiological aspect of the nerve and the decrease in the interosseous nerve in the CSA (5 mm^2^) (Figure 3D), with a partial improvement in forearm-muscle strength.

## 4. Discussion

Although it is listed as a rare disease, PTS appears to be more frequent than previously thought. Clinical investigation, EMG, and NCS remain valid tools for the diagnosis of this disease, but this could benefit greatly from NUS. In a large number of papers considered in our review, ultrasonography was the only diagnostic investigation used, and, moreover, in those cases in which the ultrasonographic study was followed by surgical exploration, it was confirmed what was seen on ultrasonography (Table 2). The great advantage of nerve ultrasonography in PTS consists in the possibility of evaluating the morphological changes in the nerves with high accuracy, localizing the lesion carefully, and providing information that is potentially useful for therapy and, in particular, for surgical approaches. It is likely that, with the improvement of the probes used, we fascicle US, and not only nerve ultrasound, will be increasingly applied. This is consistent with our previous observation about fascicle involvement [37]. A proposed classification recognized four different degrees of lesion severity and, according to this classification, lesions of grades III and IV are considered pathognomonic of PTS, and surgical treatment is indicated for such lesions. Instead, grade I and II lesions are nonspecific and may benefit from conservative treatment [14]. Nevertheless this remains a matter for debate. Although Arányi’s classification appears to be the most widely used and comprehensive, there remains an important difficulty in understanding its validity, both because of the low number of patients in his case series and because of the quality of studies currently in the literature. An important aspect that emerges from our review and from the evaluation of our patients is that, although among the old definitions of PTS we find “plexitis” of the brachial plexus, in reality, the involvement of nerves outside the plexus appears more frequent [21]. Therefore, it is accurate describe “multifocal fasciculitis”, and, sometimes, as in our case 1, even “multiple” fasciculitis is possible and, for that reason, it is necessary to explore the entire length of the suspected nerve. In a recent review, Gstoettner et al. [10] developed a flowchart for a diagnostic and therapeutic approach for when PTS is suspected. They declared that when the symptomatology is compatible with a diagnosis of PTS, it is necessary to start with conservative and symptomatic treatment and, if there is no resolution after 3 months, it is necessary to perform instrumental investigations, such as NUS and MRI, to evaluate the possibility of surgical treatment. Surgical or conservative treatments depend on the severity of the lesion, and surgical approaches deal with intrafascicular neurolysis or neurorrhaphy/grafting, while conservative treatment is usually based on steroid therapy [10].

In our experience, although we confirmed the variability of the prognoses, we believe that it is important to perform an imaging study on the first visit, to have as much information as possible for the choice of treatment. Another question that remains unresolved is the interpretation of the prognostic power of ultrasound changes. It is not yet clear how ultrasound presentation can help us to predict outcomes because of the poor research on the subject and the small number of patients whose ultrasound appearance has been studied by correlating it with prognosis. Indeed, although there is a tendency to confirm a negative prognosis in cases of hourglass-like pattern or fascicular entwinement, in some cases, there is spontaneous resolution without the need for surgical treatment, as described in case 2. However, when gross and multiple alterations are revealed, the prognosis appears worse and a surgical approach may be proposed as soon as possible, as occurred in case 1. The study of lesions, the degree of nerve constriction, and the number of rotations are essential information in order to assess the anatomical severity of damage that is likely to be related to worse outcomes, and to evaluate targeted interventional procedures.

## 5. Conclusions

In conclusion, it is still not clear whether PTS represents a unique entity or a group of disorders that differs depending on nerve involvement, severity, and prognosis. Our review shows that PTS is a pathology that more frequently involves the peripheral nerve rather than being exclusive to the brachial plexus, as previously claimed. For these reasons, if this clinical condition is suspected, we suggest exploring with the entire nerve with US. We hope that the widespread diffusion of NUS in PTS in the diagnostic phase and monitoring will make it possible to obtain more information about the prognoses of different ultrasound patterns, so that clearer therapeutic indications can be given.

## Figures and Tables

**Table 1:** Articles analyzed to perform the review containing information on NUS and other instrumental investigations.

	Author	Study	Number of Patients	Number of Controls	EMG	MRI	US
1	Arányi Z, 2015 [14]	Retrospective cohort	14		-	x	x
2	Lieba-Samal D, 2016 [17]	Retrospective cohort	6		x	x	x
3	Akane M, 2016 [9]	Retrospective cohort	49		x	x	x
4	Abraham A, 2016 [24]	Case series	2		-	-	x
5	Danielson LM, 2021 [25]	Letter to the Editor	1		x	-	x
6	Gruber L, 2017 [22]	Retrospective cohort	14	15	x	x	x
7	Sneag DB, 2017 [11]	Retrospective cohort	6		x	x	x
8	Nakagawa Y, 2018 [26]	Case report	1		-	x	x
9	Noda Y, 2017 [16]	Case series	5		x	x	x
10	Arányi Z, 2017 [15]	Retrospective cohort	53		x	x	x
11	Sneag DB, 2018 [21]	Retrospective cohort	27		-	x	x
12	Su PH, 2019 [22]	Case report	1		-	-	x
13	Porambo ME, 2019 [27]	Case report	1		x	x	x
14	van Rosmalen M, 2019 [20]	Retrospective cohort	51	50	-	x	x
15	Gstoettner C, 2020 [10]	Review	-		x	x	x
16	Krishnan KR, 2021 [28]	Retrospective cohort	24		x	x	x
17	Arányi Z, 2020 [29]	Case series	3		x	x	x
18	Krishnan KR, 2020 [30]	Case report	1		x	x	x
19	Kim MG, 2019 [31]	Case report	1		x	x	x
20	Van Alfen N, 2017 [18]	Editorial	-		x	x	x
21	Kele H, 2014 [32]	Case report	1		x	x	x
22	Decard BF, 2015 [33]	Case report	1		x	x	x
23	Becciolini M, 2022 [34]	Response to letter to Editor	0		x	-	x
24	Zanette G, 2020 [35]	Case series	2		x	x	x
25	Wang Y, 2019 [36]	Retrospective	20		x	-	x
26	Peng Wu, 2014 [8]	Retrospective	41		x	-	x

**Table 2:** The figure shows the numerical data on the nerve alteration found in the works considered and the methods used.

	Total Patients Studied	Method	Focal, Multifocal, or Diffuse Enlargement	Focal Incomplete Constriction	Focal Complete Constriction and Hourglass-Like Appearance	Fascicular Entwinement	Stiffness	Color Alteration
Arányi Z, 2015 [14]	14	NUS	57% (8/14)	36% (5/14)	50% (7/14)	28% (4/14)		
Lieba-Samal D, 2016 [17]	6	NUS	100% (6/6)					
Akane M, 2016 [9]	19/51	Surgery	21% (4/19)	38% (8/19)	5% (1/19)		38% (8/19)	36.8% (7/19)
Abraham A, 2016 [23]	2	NUS	100% (2/2)					
Danielson LM, 2021 [24]	1	NUS	100% (1/1)			100% (1/1)		
Gruber L, 2017 [25]	14 patients 15 controls	NUS	100% (14/14)	0% (14/14)	0% (14/14)			
Sneag DB, 2017 [11]	6	MRI and Surgery			100% (6/6)			
Nakagawa Y, 2018 [26]	1	MRI	100% (1/1)		100% (1/1)			
Noda Y, 2017 [16]	5	NUS	100% (5/5)		60%(3/5)			
Arányi Z, 2017 [13]	53	NUS	43% (23/53)	17% (9/53)	14% (7/53)	9% (4/53)		
Sneag DB, 2018 [21]	38	NUS			84% (32/38)			
Porambo ME, 2019 [27]	1	NUS	100% (1/1)	100% (1/1)				
van Rosmalen M, 2019 [20]	50 patients 51 controls	NUS	100% (50/50)					
Krishnan KR, 2021 [28]	24	MRI and NUS			100% (24/24)			
Arányi Z, 2020 [29]	3	NUS	66% (2/3)		100% (3/3)			
Krishnan KR, 2020 [30]	1	MRI, Surgery and NUS	100% (1/1)					
Kim MG, 2019 [31]	1	NUS and Surgery	100% (1/1)	100% (1/1)				
Kele H, 2014 [32]	1	NUS	100% (1/1)					
Decard BF, 2015 [33]	1	NUS	100% (1/1)					
Zanette G, 2020 [35]	2	NUS	100% (2/2)					
Wang Y, 2019 [36]	20	NUS performed on 1 of 20 patients			5% (1/20)			
Peng Wu, 2014 [8]	41	24 Surgery 17 NUS	66% SURGERY (16/24)		100% SURGERY 100% NUS (41/41)			

## Data Availability

Data supporting the results are not available.
